# L1000 connectivity map interrogation identifies candidate drugs for repurposing as SARS-CoV-2 antiviral therapies

**DOI:** 10.1016/j.csbj.2020.11.054

**Published:** 2020-12-06

**Authors:** Wezi Sendama

**Affiliations:** a Translational and Clinical Research Institute, Newcastle University, Newcastle upon Tyne, United Kingdom; b The Newcastle upon Tyne Hospitals NHS Foundation Trust, Newcastle upon Tyne, United Kingdom

**Keywords:** Drug repurposing, Connectivity map, Drug screening, Coronavirus, Antiviral, TMPRSS2

## Abstract

Adaptive clinical trials are underway to determine the efficacy of potential therapies for COVID-19, with flexibility to include emerging therapies if there is sufficient preclinical evidence for their potential utility. *In silico* screening of connectivity maps, which link gene expression profiles to libraries of perturbagens, may facilitate the identification of such emerging therapies. The L1000 Connectivity Map is built from samples of transcripts taken from gene expression profiles of cells in various experimental conditions followed by computational inferences of the remainder of the transcriptome. Searching the L1000 Connectivity Map for modulators of a protease that facilitates coronavirus infection identifies plausible candidate drugs for repurposing as antiviral agents against SARS-CoV-2 following further investigation.

The pace of propagation of the COVID-19 pandemic has necessitated a search for effective therapeutics for the disease. Adaptive clinical trials such as the Randomised Evaluation of COVID-19 Therapy (RECOVERY) trial (ISRCTN number ISRCTN50189673; ClinicalTrials.gov identifier NCT04381936; https://www.recoverytrial.net/) have facilitated the evaluation of plausible therapies while remaining flexible to incorporate study arms for emerging therapies as and when preclinical studies suggest their efficacy. The design of the RECOVERY trial in particular allows for its Trial Steering Committee to amend its protocol to include or exclude treatment arms in response to new evidence.

Computational screening of libraries of drugs and tool compounds may help to identify compounds with the potential to treat COVID-19. The translation of such compounds to the clinic may be accelerated if they are identified from libraries of drugs that have prior approval for other indications and therefore have known safety and interaction profiles. The L1000 Connectivity Map provides such a platform for *in silico* screening by cataloguing a sample of the gene expression profiles of cancer cell lines exposed to a library of perturbagens, with computational inference of the remainder of the transcriptomes with acceptable accuracy on the basis of the 978 measured transcripts [1]. Using data from the more limited predecessor to the L1000 Connectivity Map, Dudley and colleagues were able to repurpose the anticonvulsant medication topiramate to treat a rodent model of inflammatory bowel disease where topiramate had not previously been described to have efficacy for such an indication [2].

COVID-19 is caused by the severe acute respiratory syndrome coronavirus 2 (SARS-CoV-2), which infects a target cell by engaging its spike (S) protein with the host cell receptor ACE2 and subsequently fusing its membrane with that of the cell. In order for the virion to enter the target cell, the S protein must be primed by the cellular serine protease TMPRSS2, the inhibition of which has been demonstrated to limit SARS-CoV-2 infection of a human lung cancer cell line *in vitro* [3]. Given this, I hypothesised that some of the perturbagens in the L1000 library would have caused a reduction in expression of *TMPRSS2* mRNA in the tested human lung cancer cell line, and furthermore, some of the identified perturbagens might be drugs with prior approvals for use in humans, meaning that they could plausibly be repurposed for use as sole or adjunctive antiviral therapies for COVID-19 after further *in vitro* and *in vivo* evaluation.

I searched the L1000 database using Genevestigator v8.0.1 (Nebion AG, Zürich). Genevestigator is a computer software platform that incorporates a suite of data analysis tools and a search engine for public high throughput functional genomics data that has been curated, quality controlled, annotated and normalised by the Nebion AG team to facilitate *meta*-analysis [4]. A normalised and annotated version of the L1000 Connectivity Map dataset is available on the Genevestigator platform, derived from the publicly available data which can be downloaded from the Gene Expression Omnibus (GEO) online database (http://www.ncbi.nlm.nih.gov/geo/) under accession number GSE70138. The significance of differentially expressed genes in data was tested in the software using the Linear Models for Microarray data (Limma) algorithm to perform a moderated *t*-test on the normalised data [5]. As an exploratory analysis of the dataset, no corrections were undertaken for multiple comparisons [6]. Genes were considered significantly differentially expressed with at least an absolute log_2_ ratio change > 0.58 (roughly 1.5-fold change) to *p* < 0.05. This is an arbitrary threshold selected for screening of the connectivity map due to precedent for 1.5-fold change being considered differentially expressed in other published microarray experiments, and also because differential expression thresholds at that level are likely to represent a good trade-off between allowing the rejection of background noise and identifying biologically meaningful changes [7].

Of the compounds in the curated dataset that were tested in 24-hour perturbation assays, 40 drugs and tool compounds were identified that significantly downregulated *TMPRSS2* expression in A549 human lung epithelial adenocarcinoma cells in at least one of the tested drug concentrations. Of these, 9 are drugs with prior approvals for use in humans for alternative indications: alpelisib/BYL719, crizotinib, fedratinib/TG-101348, neratinib, nilotinib, nintedanib, ruxolitinib, selumetinib, and vemurafenib (Table 1). (It should be noted that the study design is suboptimal to discern dose-related *TMPRSS2* expression changes.) None of these compounds significantly affected *ACE2* expression. On the basis of literature searches, ruxolitinib and selumetinib are drugs of particular interest.

**Table 1:** Change in TMPRSS2 expression in A549 cells exposed to perturbagens or DMSO control for 24 h. All listed drugs are approved by the United States Food and Drug Administration for various clinical indications. Asterisk (*) denotes p < 0.05. Dagger (†) denotes data only available from n = 2 experiments; n = 3 for all other experiments. DMSO = dimethyl sulfoxide; PI3K = phosphatidylinositol 3-kinase; ALK = anaplastic lymphoma kinase; JAK = Janus kinase; MEK = mitogen-activated protein kinase kinase.

Perturbagen	Drug class	Concentration (µM)	log_2_ ratio change in *TMPRSS2* expression (perturbagen/DMSO)	p value
Alpelisib/BYL719	PI3K inhibitor	0.04	−0.94	0.012*
		0.12	−0.97	0.010*
		0.37	−0.69	0.068
		1.11	−0.75	0.046*
		3.33	−0.50	0.186
		10	−0.52	0.162
Crizotinib	ALK inhibitor	0.12 †	−0.32	0.485
		0.37	−0.57	0.127
		1.11	−0.44	0.243
		3.33	−0.76	0.043*
		10 †	−0.09	0.844
Fedratinib/TG-101348	JAK2 kinase inhibitor	0.04	−0.60	0.112
		0.12	−0.68	0.069
		0.37	−0.76	0.042*
		1.11	−0.65	0.083
		3.33	−0.38	0.307
		10	−0.41	0.278
Neratinib	Tyrosine kinase inhibitor	0.04	−0.29	0.441
		0.12	−0.36	0.336
		0.37	−0.60	0.109
		1.11	−0.96	0.011*
		3.33	−0.22	0.564
		10	−0.59	0.116
Nilotinib	Bcr-Abl tyrosine kinase inhibitor	0.04	−0.81	0.031*
		0.12	−0.65	0.083
		0.37	−0.60	0.112
		1.11	−0.44	0.243
		3.33	−0.56	0.138
		10	−0.12	0.754
Nintedanib	Tyrosine kinase inhibitor	0.04	−0.85	0.024*
		0.12	−0.36	0.334
		0.37	−0.42	0.263
		1.11	−0.23	0.541
		3.33	−0.38	0.316
		10	0.21	0.568
Ruxolitinib	JAK1/2 inhibitor	0.04	−0.55	0.141
		0.12	−0.84	0.025*
		0.37	−0.52	0.168
		1.11	−0.51	0.177
		3.33	−0.61	0.105
		10	−0.82	0.028
Selumetinib	MEK inhibitor	0.04	−0.31	0.402
		0.12	−0.51	0.176
		0.37	−0.82	0.030*
		1.11	−0.55	0.146
		3.33	−0.61	0.104
		10	−0.92	0.014*
Vemurafenib	B-raf inhibitor	0.04	−0.29	0.444
		0.12	−0.35	0.355
		0.37	−0.90	0.014*
		1.11	−0.72	0.054
		3.33	−0.66	0.077
		10	−0.43	0.249

Ruxolitinib is an inhibitor of Janus kinase (JAK) enzymes that has been approved by the United States Food and Drug Administration (FDA) for the treatment of myelofibrosis, a condition characterised by abnormal clonal proliferation of haematopoietic stem cells. JAKs operate as transducers downstream of several receptors for pro-inflammatory cytokines, which makes them attractive candidates for treatment of conditions in which inflammation is dysregulated, as can be the case in COVID-19 [8]. With that rationale, Cao and colleagues undertook a single-blind, randomised controlled phase 2 trial of ruxolitinib versus placebo in addition to standard of care treatment in severe COVID-19. The results are instructive but perhaps disappointing for the potential of ruxolitinib as an antiviral medication aside from its anti-inflammatory effect: there was no significant difference in the secondary outcome of time to SARS-CoV-2 clearance between ruxolitinib and placebo recipients, although the authors concede their sample size was limited [9].

Selumetinib is a mitogen-activated protein kinase (MAPK/ERK) kinase (MAPKK/MEK) inhibitor that has FDA approval for the treatment of neurofibromatosis type 1, a multisystem disorder that is most often characterised by a propensity for neurofibroma formation. Although selumetinib has not been trialled in COVID-19 patients like ruxolitinib has, *in vitro* data suggest it may have some antiviral activity. Selumetinib (as well as some other MAPK pathway modulators) was found to inhibit the infection of a human hepatoma cell line with Middle East respiratory syndrome coronavirus (MERS-CoV) [10]. MERS-CoV is a coronavirus related to SARS-CoV-2 that also requires TMPRSS2 for S protein priming before cell entry, and similarly to SARS-CoV-2, inhibition of TMPRSS2 with a serine protease inhibitor limits MERS-CoV infection of simian kidney epithelial cells *in vitro* [11]. This, in conjunction with the observation from the *in silico* screen that suggests reduced mRNA expression of *TMPRSS2* in cells exposed to selumetinib, identifies the MEK inhibitor as a plausible candidate for further evaluation in experimental models relevant to COVID-19.

*TMPRSS2* is under the transcriptional control of the androgen receptor (*AR*) [12], which translocates to the nucleus to facilitate transcription of its target genes upon binding by androgens [13]. The expression of *AR* itself is regulated by the transcription factor CREB1, which is activated by phosphorylated MAPK. MEK inhibitors such as selumetinib (because they are inhibitors of MAPK kinase) are therefore able to reduce the expression of AR *in vivo* [14], which may consequently reduce the transcription of AR target genes (including *TMPRSS2*). It is plausible that reduced TMPRSS2 availability as a result of MEK inhibition would limit SARS-CoV-2 cell entry, but this hypothesised mechanism would need to be validated experimentally. Androgen regulation of *TMPRSS2* expression may also provide a mechanistic explanation for the observation that male sex is associated with a higher COVID-19 case fatality rate than female sex [15], but again, experimental validation would be required.

Adaptive clinical trials like the RECOVERY trial will be pivotal in gaining control of the COVID-19 pandemic and candidate compounds for inclusion in such trials will need to be identified with sound scientific rationale. Connectivity maps such as L1000 are likely to prove essential in that regard, enabling researchers to screen for drugs that may be repurposed for new indications. Screening of the L1000 Connectivity Map in this case identifies selumetinib as a plausible antiviral candidate to treat COVID-19, but it is important to note that such *in silico* screening is an aid and a precursor to further *in vitro* and *in vivo* evaluation, not a replacement for it.

## CRediT authorship contribution statement

**Wezi Sendama:** Conceptualisation, investigation.

## Declaration of Competing Interest

The authors declare that they have no known competing financial interests or personal relationships that could have appeared to influence the work reported in this paper.
